# Toxicity and Antioxidant Activity of Fullerenol C_60,70_ with Low Number of Oxygen Substituents

**DOI:** 10.3390/ijms22126382

**Published:** 2021-06-15

**Authors:** Ekaterina S. Kovel, Arina G. Kicheeva, Natalia G. Vnukova, Grigory N. Churilov, Evsei A. Stepin, Nadezhda S. Kudryasheva

**Affiliations:** 1 Institute of Biophysics SB RAS, FRC KSC SB RAS, 660036 Krasnoyarsk, Russia; n-qdr@yandex.ru; 2 Institute of Physics SB RAS, FRC KSC SB RAS, 660036 Krasnoyarsk, Russia; nata_hd@rambler.ru (N.G.V.); churilov@iph.krasn.ru (G.N.C.); 3 FRC KSC SB RAS, 660036 Krasnoyarsk, Russia; khyzylsyg@mail.ru; 4 Institute of Fundamental Biology and Biotechnology, Siberian Federal University, 660041 Krasnoyarsk, Russia; stepin-kirill@mail.ru

**Keywords:** fullerenol, toxicity, antioxidant activity, reactive oxygen species, bioluminescent assay, hormesis

## Abstract

Fullerene is a nanosized carbon structure with potential drug delivery applications. We studied the bioeffects of a water-soluble fullerene derivative, fullerenol, with 10-12 oxygen groups (F10-12); its structure was characterized by IR and XPS spectroscopy. A bioluminescent enzyme system was used to study toxic and antioxidant effects of F10-12 at the enzymatic level. Antioxidant characteristics of F10-12 were revealed in model solutions of organic and inorganic oxidizers. Low-concentration activation of bioluminescence was validated statistically in oxidizer solutions. Toxic and antioxidant characteristics of F10-12 were compared to those of homologous fullerenols with a higher number of oxygen groups:F24-28 and F40-42. No simple dependency was found between the toxic/antioxidant characteristics and the number of oxygen groups on the fullerene’s carbon cage. Lower toxicity and higher antioxidant activity of F24-28 were identified and presumptively attributed to its higher solubility. An active role of reactive oxygen species (ROS) in the bioeffects of F10-12 was demonstrated. Correlations between toxic/antioxidant characteristics of F10-12 and ROS content were evaluated. Toxic and antioxidant effects were related to the decrease in ROS content in the enzyme solutions. Our results reveal a complexity of ROS effects in the enzymatic assay system.

## 1. Introduction

Fullerenes are carbon nanomaterials known for their unique cage structure. The first representative of the fullerene group was discovered by Sir Harold Kroto and his group in 1985 [1,2]. The discovery and structural study of fullerene [1,3] pioneered the new field of carbon allotropes: fullerene chemistry. This new field provided various fullerene derivatives [4,5,6] with potential features useful for numerous applications.

Some of the unique properties of fullerene are its three-dimensional structure and chemical reactivity [7,8,9,10,11,12,13,14,15]. Numerous fullerene-based compounds with different biological targets have been synthesized; biomedical and bioengineering aspects for their application are currently under intensive review [4,16,17,18,19,20,21]. Fullerenes are prospective candidates for anticancer or antimicrobial therapy, cytoprotection, enzyme inhibition, controlled drug delivery, contrast-based or radioactivity-based diagnostic imaging, radio-protection, photosensitization, and biomimetics. Fullerene properties such as antioxidant or pro-oxidant potential, toxicity, membranotropicity, protein-binding affinity, antiviral, antimicrobial, and anti-apoptotic ability are currently under investigation.

The most common form of fullerene involves 60 atoms of carbon arranged in a spherical structure, with every carbon atom forming a bond to three other adjacent carbon atoms through sp^2^-hybridization [1,3]. The highly conjugated π-electron system of the fullerene carbon cage provides it with electron-acceptor capacities and, hence, with catalytic activity in multiple biochemical processes as well as antiradical activity [7,8,9,10,14,15,16,17]. The pristine C_60_ is extremely hydrophobic and insoluble in water and biological media, which hampers its utilization in biomedical and pharmaceutical applications [22,23]. A conventional method used to prepare a water-soluble form of fullerene C_60_ (hydrated fullerene C_60_) involves ultrasonic treatment of fullerene water dispersions [23]. Andrievsky et al. supposed hydrated fullerene C_60_ to be a hydrophilic supramolecular complex consisting of a fullerene C_60_ macromolecule enclosed in a hydrate shell [22]. Many concerns continue to accumulate regarding the beneficial effects of hydrated fullerene C_60_ that tend to emerge at extremely low concentrations [23].

Additionally, an increase in water solubility can result from the modification of fullerene’s surface with polar chemical groups. A variety of fullerene C_60_ derivatives with various polar functional groups have been developed [4,24]. The fullerene C_60_ derivatives, fullerenols C_60_(OH)_n_, which involve multiple hydroxyl groups chemically bound to the surface of the C_60_ skeleton, have been synthesized [25,26]. The water-soluble C_60_ modifications expand their biomedicine applications, such as neuroprotection, drug and gene delivery, protection from radiation-induced injury, photosensitization, sonosensitization, bone-repair, and biosensing [27].

Biological activity of C_60_-fullerenols with varying numbers of oxygen groups have been extensively studied in previous decades [28,29,30,31]. Nevertheless, most experimental studies did not provide proper conditions to compare biological activities of different fullerenols. Comparable conditions were provided in [25]: Eropkin et al. studied biological activity of fullerenols C_60_(OH)_12-14_, C_60_(OH)_18-24_, and C_60_(OH)_30-38_. In vitro toxicity of hydroxylated fullerenes in human skin cells was assessed and explained by an increase in the number of hydroxyl groups from 20 to 32 [32]. Comparable experimental conditions were provided using bioluminescence assay systems, unicellular, and enzymatic [33]; they revealed lower toxicity and higher antioxidant activity of fullerenols with fewer oxygen substituents (24–28) than compared to a greater number of the substituents (40–42).

Although fullerene derivatives have been demonstrated to act as strong antioxidants in water solutions, evidence regarding their antioxidant properties are controversial. Some studies confirm the presence of an antioxidant effect of fullerenes, whereas others demonstrate oxidative stress in cellular systems [34]. For example, in [35], cell-based in vitro bioassays were used to compare antioxidant activity and toxicity of fullerenols with varying numbers of hydroxyl groups.

Bioluminescence-based assays are appropriate candidates to study and compare biological activity of fullerene derivatives due to their simplicity, sensitivity, and high rates of analysis (1–10 min). Bioluminescent assays use luminescence intensity as a physiological testing parameter; this parameter can be easily measured using simple physical devices. These advantages allow investigators to conduct a large number of tests under comparable conditions during a short time-period; therefore, these tests are adapted to extensive statistical processing, particularly, at low-concentration (low-intensity and low-dose) exposures, which usually produces “noisy” responses and are described in terms of “stochasticity”.

The bioluminescence bacterial assay is commonly used; it has been applied for more than fifty years to monitor a general toxicity of complex media [36,37,38,39,40]. The use of the bioluminescence enzymatic assay is a relatively new direction in the toxicology practice [41]. As a rule, the enzymatic bioluminescent assay is based on two coupled enzymatic reactions of luminous bacteria (presented below in Section 3.2, Material and Methods). This assay can be used to assess the “general” toxicity of test samples, which is similar to the cellular bioassay. This type of toxicity integrates all interactions of the bioluminescent assay system with toxic compounds: redox processes, polar and non-polar binding, etc. Furthermore, the enzymatic assay is specific to oxidizers since its additional kinetic parameter, induction period, depends on the redox potentials of toxic compounds [42]. Therefore, the enzymatic assay can be additionally applied to evaluate the “oxidative type” specific toxicity, which is attributed to the redox properties of toxic compounds only. Due to its ability to characterize toxicities of “general” and “oxidative” types, this enzymatic assay can reveal the ability of fullerene derivatives to intermediate redox reactions as well as evaluate their ability to inhibit/stimulate biochemical processes via surface hydrophobic/hydrophilic interactions.

Previously [43,44], the bioluminescent enzymatic assay system was used to evaluate toxicity in solutions of organic and inorganic oxidizers (i.e., quinones and polyvalent metals, respectively) as well as to study changes of toxicity of general and oxidative types under exposure to humic substances [45,46,47]. Later, toxicity and antioxidant activity of a series of fullerenols of different structures were evaluated and compared in [33,48,49,50,51,52]. It should be mentioned that antioxidant effects of fullerenols were found at low and ultralow concentrations (<0.001 g L^−1^). It was shown in [53] that the bioluminescent enzymatic assay system can be additionally applied to monitor pro-oxidant effects of bioactive compounds.

Based on our previous results, we suggested [33] that an increase in the number of hydroxyl groups decreases fullerenol’s ability of reversible radical trapping due to the reduction in the size of the available conjugated π-electron system. Following this logic, the fullerene with the lowest number of oxygen groups and, hence, largest size of conjugated π-electron system should be characterized with the lowest toxicity and highest antioxidant activity. The current study aims to verify this suggestion under comparable experimental conditions. The first goal of our study is to evaluate the antioxidant activity and toxicity of fullerene with 10–12 oxygen groups relative to fullerenols with identical carbon cage structure but higher number of oxygen groups: 24–28 or 40–42.

The second goal of our study is concerned with the evaluation of the role of Reactive Oxygen Species (ROS) in toxic and antioxidant effects of fullerenols; a fullerenol with 10–12 oxygen groups was used as a representative of the group of fullerene derivatives.

It is known [54] that the chemical structure of fullerene derivatives permits them to neutralize ROS effectively. This process can take place in all media: solutions of low-molecular-weight and high-molecular-weight compounds, biomolecules, cells, and tissues. From the other point of view, healthy cells are known to produce ROS as a consequence of physiological processes, which can be localized in the nucleus, cytoplasm, or cell membrane. ROS are active in cell cycle regulation and signaling, proliferation, apoptosis, regulation of kinase activity, and gene expression [55]. It is supposed that excess amounts of ROS results in oxidative stress, DNA damage, and cell death [56,57,58]. Some studies indicate activation of physiological functions at low ROS concentrations and their inhibition at high ROS concentrations [59,60]. The studies mentioned used suspensions of luminous marine bacteria as a bioassay and hydrogen peroxide as a representative of ROS.

In general, toxicological investigations should use a physico-chemical approach based on the relationships between structures of exogenous compounds and their attendant toxic/adaptive responses. This approach forms a basis for the selection of compounds with characteristics that are suitable for biomedical and pharmaceutical purposes. Correlations between physico-chemical characteristics of bioactive compounds and their bioeffects are the most desirable result of these investigations. Therefore, a simplification of assay systems and active particles is of fundamental toxicological interest, which justifies our application of enzymatic reactions as assay systems and consideration of ROS as indicators of toxicity-based primary interactions.

Our study correlates toxic/antioxidant properties of the fullerenol with ROS content in enzymatic assay solutions.

## 2. Results and Discussion

We analyzed toxicity and antioxidant activity of fullerenol F10-12, a representative of a fullerenol group. Its characteristics were compared to those of two other fullerenols with similar carbon cage structures but with a higher number of oxygen substituents (24–28 and 40–42). The latter two fullerenols were studied previously under comparable conditions [33]. Short abbreviations for the fullerenols are introduced in Table 1.

### 2.1. Fullerenol Toxicity

We examined the toxicity factor of F10-12 using an enzymatic bioluminescence assay. The application of bacterial bioluminescent enzymatic assays for toxicity monitoring was previously justified in [41,45]. Suppression of bioluminescence intensity was considered as evidence of fullerenol toxic effects at an enzymatic level. This suppression is associated with the inhibition of chemical and biochemical processes by low-molecular or nano-compounds as previously discussed [45,48,50,61].

**Figure 1:**
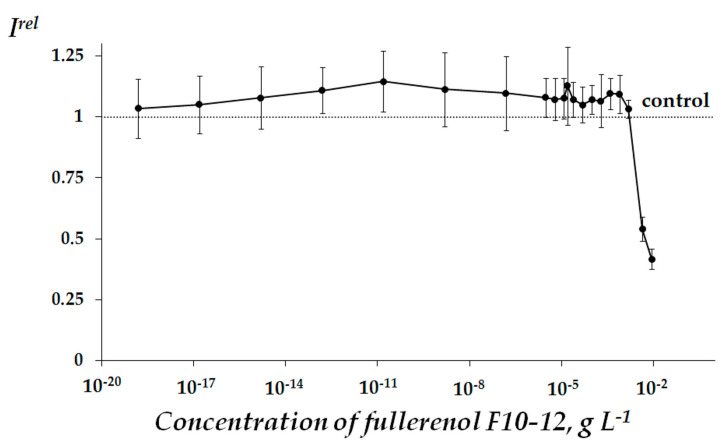
Bioluminescence intensity of enzymatic system, *I^rel^*, at different concentrations of fullerenol F10-12.

Dependency of relative bioluminescence intensity *I^rel^* (Equation (1), Section 3.2) on the concentration of F10-12 was obtained (Figure 1). It is observed that F10-12 suppresses bioluminescence of the enzymatic assay at concentrations >0.002 g L^−1^ (*p* < 0.05). The value of *EC_50_* for F10-12 was determined as 0.006 g L^−1^. A slight activation effect at low concentrations of F10-12 (<0.002 g L^−1^) can be observed in Figure 1 for average values of *I^rel^*; however, this was not statistically confirmed in the current experiment (*p* > 0.05).

The results were compared to the two fullerenols, F24-28 and F40-42, studied earlier [33]. Their *EC_50_* values were determined as 0.021 and 0.007 g L^−1^, respectively. Hence, toxicity of F10-12 (i.e., fullerenol with lower number of oxygen substituents) is higher than that of F24-28 and comparable to that of F40-42.

Therefore, the bioluminescence enzymatic assay demonstrated that the toxicity of fullerenols is not a simple function of the number of oxygen substituents since the toxicity of F10-12 is not the lowest one among the studied fullerenols. It is likely that the other characteristics of the fullerenols, such as water solubility, are responsible for the observed toxicity of F10-12 measured with the enzymatic assay. This supposition is supported by theoretical studies in fullerenol’s solubility. It was found that the average number of fullerenol-water hydrogen bonds increases in proportion to the number of hydroxyl groups between 12 and 24 [62], which provides higher solubility. The other authors [31] predicted that grafting more than 36 hydroxyl groups per C-60 cage is unlikely to be useful; hydration of C_60_(OH)_44_ is less effective, despite the larger number of hydroxyl groups. It was concluded that the involvement of 36–44 hydroxyl groups to the structure of fullerenols results in effective intramolecular interactions of OH-groups, conflicting with the hydrogen bonds and with the solvent. Toxic and antioxidant effects of fullerenols were predicted based on these results.

**Figure 2:**
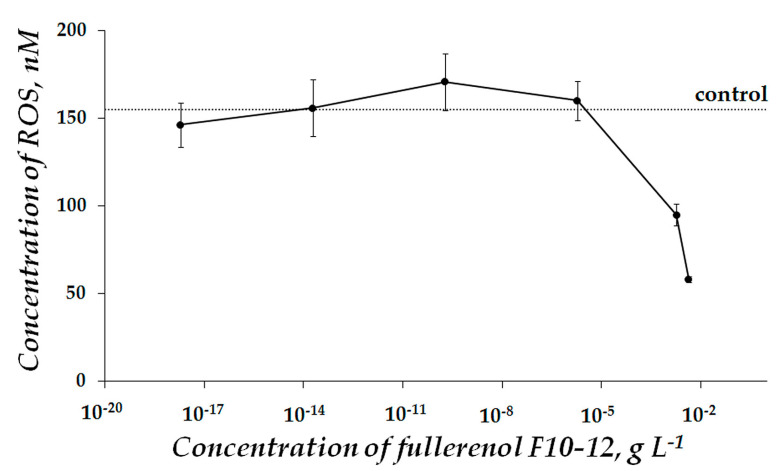
Concentration of ROS in the enzymatic assay system at different concentrations of fullerenol F10-12. Incubation time—45 min. ROS concentration in the control sample is 155 nM.

In order to verify the role of ROS in the toxic effect of F10-12 (Figure 1), we determined ROS content under conditions of the experiment (Figure 2). Correlations between bioluminescence intensity *I^rel^* and ROS concentration in solutions of different F10-12 concentrations (Figure 1 and Figure 2) were analyzed and the correlation coefficient, r, was calculated as 0.86. This result reveals a high positive correlation (*r* > 0.7) between *I^rel^* and ROS concentration. This correlation suggests that F10-12 toxicity can result from the lack of ROS in the bioassay solution under its addition. 

The physicochemical mechanism of fullerenol’s toxic influence on the enzymatic assay system is likely due to its ability to neutralize free radicals [33], including peroxide radicals. It is known that one of the intermediates of the bioluminescent luciferase’s reaction (reaction 2, Section 3.2), flavin peroxy-semiacetal [63,64], is a peroxide that is categorized as a ROS. Hence, the decrease in ROS content at high fullerene concentrations can account for the inhibition of the bioluminescent reaction (reaction 2, Section 3.2). It was discussed earlier [65] that the oxygen-detoxifying function promoted the evolutionary emergence of a series of bioluminescence systems, including the bioluminescence systems of marine bacteria. The bacterial bioluminescence reaction (reaction 2, Section 3.2) is applied as a model of enzymatic oxygen-dependent reactions taking place in all living organisms.

In summary, the toxic effect of F10-12 is a result of ROS decrease in the enzymatic bioassay solutions. It takes place at high fullerenol concentrations (>0.002 g L^−1^) and demonstrates antiradical properties of the fullerenol.

The result suggests that a lack of ROS can cause a toxic effect. Therefore, the previous results on the suppression of antiviral activity [25] and the decrease in bioluminescence intensity of bacteria-based [33,49,51] and enzyme-based [32,46,47,48,49] assays can also be explained by ROS neutralization in high-concentration fullerenol solutions.

Our results improve our understanding of ROS functions in biological systems: both an excess and a lack of ROS can suppress biological processes. As previously mentioned, it is commonly recognized that only the excess of ROS leads to oxidative stress and toxic effects through DNA damage and cell death [56,57,58]. Hence, there likely exists an optimum interval of ROS concentrations for living systems.

Based on the results presented in Figure 1, we chose the range of non-toxic concentrations (<0.002 g L^−1^) for further low-concentration experiments to study the antioxidant activity of F10-12 (Section 2.2.2 below).

### 2.2. Fullerenol Antioxidant Activity

Fullerenol’s antioxidant activity was studied in model solutions of oxidizers as previously utilized [44,46,47,48]. Oxidizers of organic or inorganic types including 1,4-benzoquinone or potassium ferricyanide (i.e., K_3_[Fe(CN)_6_]) were used [42,66,67]. Standard redox potentials of the oxidizers chosen are high: 0.71 V and 0.36 V, respectively. The exposure of enzyme reactions to the oxidizers might be considered as a simplified model of “oxidative stress” at the molecular level.

Quinone and iron (III) are chosen here as important representatives of intra-cellular and extra-cellular oxidizers. Quinones are produced environmentally as a result of oxidative transformation of phenols and occupy the third position in the list of top widespread pollutants (after oil products and metal salts) [68]. Phenolic substances are also synthesized by soil bacteria as molecular signaling molecules in microbial communication and as adaptogens [69] and induce redox transformations in soils and aquifers, especially at low pH in the presence of iron (III) [70,71].

Two bioluminescence kinetic parameters were monitored in the presence of the oxidizers: bioluminescence intensity (*I*) and bioluminescence induction period (*T*). The antioxidant coefficients *I^rel^_Ox_* and *T^rel^_Ox_* were then calculated (Equations (2) and (3), Section 3.2). Changes in toxicity of general and oxidative type were evaluated using these parameters, respectively. It is supposed that oxidative toxicity is a function of redox activity of toxic media, while general toxicity is based on complex processes involving redox and polar/apolar interactions in the enzyme system. The data obtained earlier [33,50,53] showed higher sensitivity of *T^rel^_Ox_* to redox effects.

#### 2.2.1. Change of General Toxicity under Conditions of Oxidative Exposure

**Figure 3:**
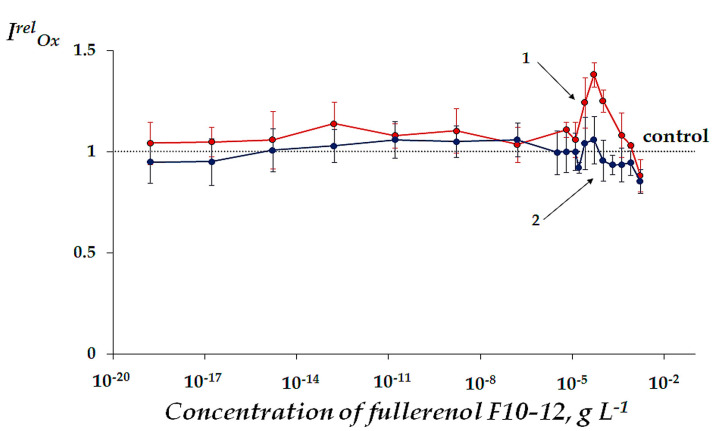
Antioxidant coefficients *I^rel^_Ox_* vs. concentration of fullerenol F10-12 in solutions of (1) 1,4-benzoquinone at *EC_50_* = 10^−5^ M and (2) K_3_[Fe(CN)_6_] at *EC_50_* = 10^−4^ M.

The bioluminescence intensity of the enzymatic system (*I*) was measured in model solutions of oxidizers 1,4-benzoquinone and K_3_[Fe(CN)_6_] at *EC_50_* in the absence and presence of F10-12. Concentration of F10-12 varied in a wide range as shown in Figure 3. Antioxidant coefficients *I^rel^_Ox_* were calculated according to Equation (2) (Section 3.2).

Figure 3 shows that F10-12 detoxified the 1,4-benzoquinone solutions (*I^rel^_Ox_* > 1) in the narrow concentration range 10^–5^–4·10^−4^ g L^−1^ (*p* < 0.05) with the maximal value of *I^rel^_Ox_* = 1.38; lower fullerenol concentrations did not significantly affect the *I^rel^_Ox_* than compared to the control (*p* > 0.05). As shown previously in similar experimental conditions [33], fullerenols with higher number of oxygen substituents, F24-28 and F40-42, also showed moderate increases in *I^rel^_Ox_* (with 1.44 and 1.44 maximal values); however, intervals of their active concentrations were wider (Table 2).

The antioxidant effects of fullerenols have been described previously [49] in terms of the hormesis model [72,73,74,75], with the specificity of nanoparticle-based colloid systems taken into consideration. The activation effect and absence of monotonic dependencies of *I^rel^_Ox_* on fullerenol concentrations [49] provided validity for the hormetic toxicological model application.

Low-concentration antioxidant and radio-protective properties of hydrated fullerene C-60 in vitro and in vivo were first identified and discussed by Andrievsky and colleagues [22]; they hypothesized catalytic-like mechanisms that determine the antiradical activity of C-60. They proposed a mechanism of action in super-small doses by means of long-range and stable water layers ordered by C-60 and is capable of the neutralization of free radicals.

It should be highlighted that only the conditions of oxidizer exposure revealed the activation of bioluminescence intensity by fullerenol (*I^rel^_Ox_* > 1), Figure 3, than compared to the non-exposed conditions, Figure 1.

Mitigation of the enzymatic response to the oxidative load was observed only in the solutions of organic oxidizer. In solutions of inorganic oxidizer, such as potassium ferricyanide (Figure 3), F10-12 did not reveal any reliable deviations of *I^rel^_Ox_* from the control (*p* > 0.05) in the entire concentration range. Similar behavior of other fullerenols, F24-28 and F40-42, was observed earlier [33]; they demonstrated an absence or negligible antioxidant effects in the solutions of the inorganic oxidizer, Table 2.

Therefore, our results demonstrate the importance of hydrophobic interactions in the redox transformations in our complex system that consists of bioluminescent enzymatic reactions, organic oxidizers, and fullerenol. Our study did not find any significant differences in antioxidant efficiencies of F10-12 and other fullerenols under similar experimental conditions. However, the valid range of activating concentrations of F10-12 in the organic oxidizer solutions was narrowed.

#### 2.2.2. Change of Oxidative Toxicity under Conditions of Oxidative Exposure

In order to monitor changes in oxidative toxicity under conditions of oxidative exposure, the bioluminescence kinetics of the enzymatic system were studied in model solutions of oxidizers 1,4-benzoquinone and K_3_[Fe(CN)_6_] at *EC_50_*. Induction periods *T* were measured in the absence and presence of fullerenol. The values of *T^rel^_Ox_* were calculated according to Equation (3) (Section 3.2).

**Figure 4:**
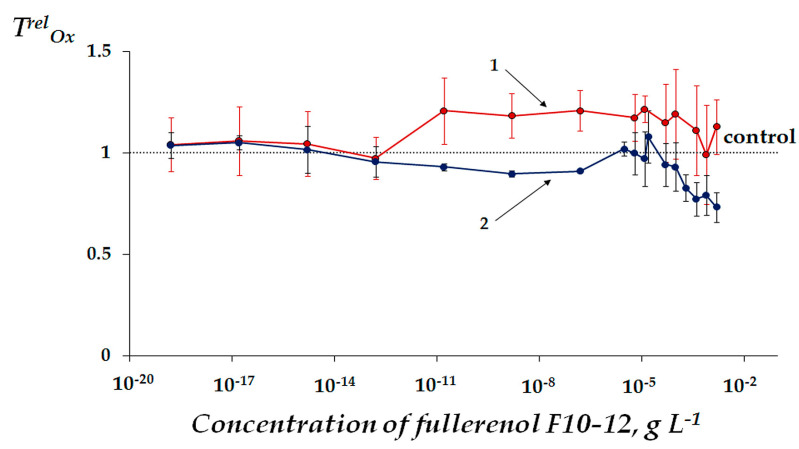
Antioxidant coefficients *T^rel^_Ox_* vs. concentration of fullerenol F10-12 in solutions of (1) 1,4-benzoquinone at *EC_50_* = 10^−5^ M and (2) K_3_[Fe(CN)_6_] at *EC_50_* = 10^−4^ M.

Figure 4 demonstrates the dependences of *T^rel^_Ox_* on the concentration of F10-12. Antioxidant effects (*T^rel^_Ox_* > 1) were found in the solutions of organic oxidizer 1,4-benzoquinone in the concentration range of 10^−13^–5·10^−5^ g L^−1^ (*p* < 0.05). However, the antioxidant effects were not evident in the solutions of inorganic oxidizer–potassium ferricyanide (*p* > 0.05): The average values of *T^rel^_Ox_* did not exceed 1.2 and 1.0, respectively. Similar to *I^rel^_Ox_*, coefficients *T^rel^_Ox_* demonstrated moderate statistically-reliable antioxidant activity of F10-12 only in solutions of the organic oxidizer.

Additionally, Table 3 shows maximal values of *T^rel^_Ox_* for F10-12 as well as for those two fullerenols with higher number of oxygen substituents, F24-28 and F40-42, studied earlier [33]. The average values of *T^rel^_Ox_* for F10-12 did not exceed 1.2, while they reached up to 1.9 for F24-28 in the organic oxidizer solutions. The data suggest that F24-28 has the highest antioxidant activity while F40-42 had the lowest one. Therefore, F10-12 is characterized by the intermediate antioxidant activity and this property of fullerenol is not a simple function of the number of oxygen substituents in the fullerene carbon cage.

Hence, our current study demonstrates that lowering the number of oxygen substituents (down to 10–12) did not increase *T^rel^_Ox_*, as is presumed in [33]. Higher efficiency of fullerenol–solvent interactions of F24-28 and related solubility in water [31] might be responsible for the high antioxidant effect of this fullerenol. Furthermore, the results support the conclusions of Eropkin et al. [25] regarding biological activity of fullerenols with different numbers of oxygen groups; the highest antiviral and protective properties were achieved by an intermediate amount of oxygen groups as opposed to the lowest variant.

The role of ROS in the detoxification of organic oxidizer solution by F10-12 was studied.

**Figure 5:**
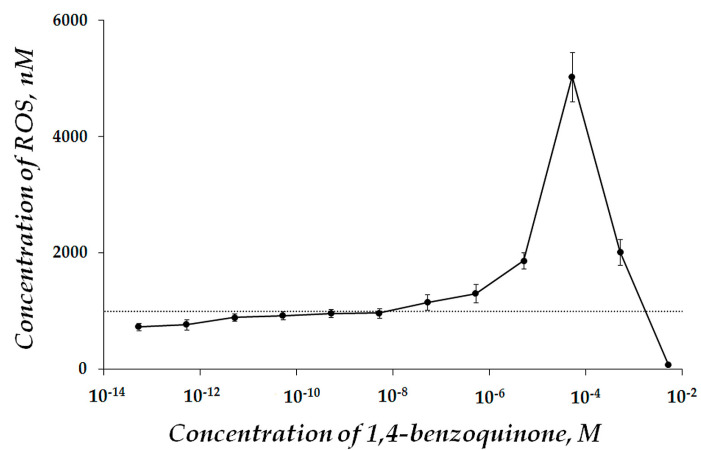
ROS content at different concentrations of 1,4-benzoquinone in water solutions. ROS concentration in control (distilled water) was 1000 nM.

Preliminarily, we evaluated ROS content in 1,4-benzoquinone water solutions. The dependence of the ROS content on 1,4-benzoquinone concentrations is presented in Figure 5. The ROS content rose by a factor of two at *EC_50_* = 10^−5^ M than compared to the control, Figure 5. Additionally, it is observed that 1,4-benzoquinone suppresses ROS at high concentrations (>10^−3^ g L^−1^). This effect was attributed earlier [76] to its ability to form dioxetanes, which is typical for unsaturated hydrocarbons [77].

**Figure 6:**
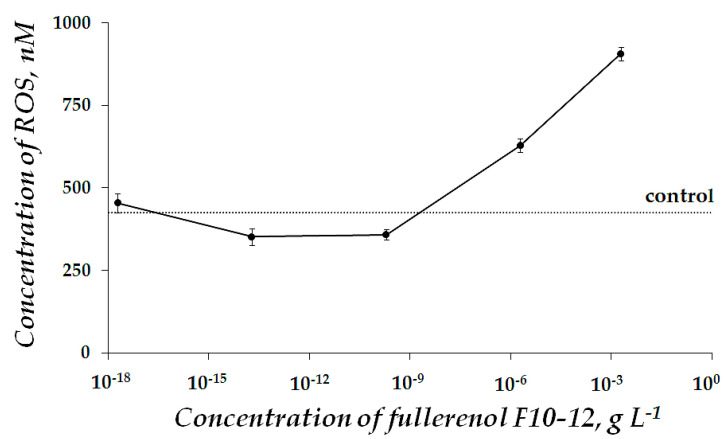
ROS content in the enzyme system in the presence of 1,4-benzoquinone (10^−5^ M) vs. concentration of F10-12. Incubation time was 15 min, pH 6,8. ROS content in the control sample was 427 nM.

Figure 6 presents the ROS content in the enzyme system at different concentrations of F10-12. A moderate decrease in ROS content (as compared to control) was observed at F10-12 concentration range of ca. 10^−17^–10^−8^ g L^−1^ (*p* < 0.05). This interval is close to the antioxidant concentration range (*T^rel^_Ox_* > 1, Figure 4, red curve). This coincidence may suggest that the antioxidant effect of F10-12 results from the lowering of ROS content in the bioassay solution under conditions of oxidizer exposure.

The correlation between *T^rel^_Ox_* and ROS concentration in solutions of F10-12 and 1,4-benzoquinone (Figure 4 red and Figure 6) was analyzed and the correlation coefficient *r* was calculated as −0.46. The value reveals a low negative correlation (0.3 < *r* < 0.7) between *T^rel^_Ox_* and ROS concentration (10^−14^–10^−3^ g L^−1^) and highlights the complexity of the processes responsible for the antioxidant effect of F10-12, which nonetheless involves ROS neutralization by the fullerenol.

Hence, we showed that fullerene F10-12 can inhibit bioluminescent enzymatic assay system or activate it. Inhibition occurs at high fullerene concentrations (>0.002 g L^–1^), whereas activation occurs at low concentrations: 10^−13^–5·10^−5^ g L^−1^ under the conditions of oxidative exposure. Both effects are concerned with ROS neutralization in solutions under the addition of fullerenol. However, the high-concentration ROS decay results in a toxic effect and the slight low-concentration ROS decay mitigates the toxic effect of the oxidizer (1,4-benzoquinone) revealing antioxidant properties of fullerenol.

## 3. Materials and Methods

### 3.1. Preparation and Characterization of Fullerenols

Carbon condensate was synthesized in the plasma of high-frequency arc discharge at atmospheric pressure [78,79]. The carbon soot included 12.6% of fullerene. The fullerene mixture (C_60_—67.4%; C_70_—16.9%; C_76_—2.9%; C_78_—2.7%; C_80_—2.1%; higher fullerenes—8%) was extracted by toluene in the Soxhlet extractor and solvent was evaporated.

Fullerenol F10-12 was obtained by dissolving the powdered mixture of fullerenes (2 mg) in benzene, then followed by transferring this solution to the aqueous phase (deionized water) by ultrasonic treatment (power 230 W, operating frequency 35 kHz) at ambient temperature and with the removal of the organic solvent [23,80].

The fullerene preparation was characterized with infrared (IR) spectroscopy [81,82] in the KBr matrix using Fourier spectrometer VERTEX 70, Bruker, Karlsruhe, Germany. The number of -OH groups was estimated by X-ray photoelectron spectroscopy (XPS) using UNI-SPECS spectrometer, SPECS Gmbh, Berlin, Germany.

IR spectroscopy (Figure S1, IR spectra of fullerenol F10-12, Supplementary Materials) showed the presence of -OH groups on the carbon skeleton. According to the sources [5,83], the stretching C=C vibration bands, evidently with different chemical microenvironments (at ~1570–1630 cm^−1^), were identified. The broad band with a maximum at 3427 cm^−1^ and those around 1390 cm^−1^ corresponds to the νO-H and δC-O-H vibrations (the latter appear also at slightly different frequencies because of the different chemical microenvironments of the carbon atoms). The carbon–oxygen moieties are characterized by the bands at 1709 cm^−1^ (νC=O) and 1090 cm^−1^ (νC-O), reflecting the presence of carbonyl (ketone) and alcohol groups. The C–O bond stretching is inevitable in all the fullerenols, which perhaps indicates the formation of hemiketal groups prior to the hydroxylation of the fullerene cage [84].

XPS was used to estimate the number of -OH groups. Gaussian/Lorentzian decomposition of the C1s line (Figure S2, XPS of fullerenol F10-12 (C1s line), Supplementary Materials) recorded binding energies of 284.8; 286.2; 289 eV, which are assigned to C-C (36%), C-O (11%) and C=O (10%), respectively [85]. The number of -OH groups calculated from the fraction of carbon atoms chemically bonded to oxygen is 21% for the sample under study. Hence, the average composition of fullerene corresponds to C_60,70_O_x_(OH)_y_, where x + y = 10–12 and y—even. Thus, the structural analysis showed that the preparation represents a fullerene derivative, which is fullerenol with 10–12 oxygen atoms.

Fullerenols C_60,70_O_y_(OH)_x_ where x + y = 24-28 (F24-28) and C_60,70_O_y_(OH)_x_ where x + y = 40-42 (F40-42) were synthesized and characterized as described in [33].

### 3.2. Bioluminescence Enzymatic Assay and Experimental Data Processing

Antioxidant activity and toxicity of fullerenols were evaluated using bioluminescence enzymatic assay, i.e., enzymatic preparation based on the system of coupled enzyme reactions catalyzed by NADH:FMN-oxidoreductase from *Vibrio fischeri* (0.15 a.u.) and luciferase from *Photobacterium leiognathid* at 0.5 mg/mL [86]. The enzyme preparation was produced at the Institute of Biophysics SB RAS, Krasnoyarsk, Russia. Antioxidant activity of F10-12 was assessed in water solutions of model oxidizers K_3_[Fe(CN)_6_] (potassium ferricyanide) and 1,4-benzoquinone. The method was established in [42,45,47,48,50,53].

The used chemicals were FMN and tetradecanal from SERVA, Heidelberg, Germany; NADH from ICN Biochemicals, Costa-Mesa, CA, USA; potassium ferricyanide from Khimreactiv, Nizhny Novgorod, Russia; 1,4-benzoquinone from Aldrich, Burlington, MA, USA. The reagents were of chemical or analytical grade.

In order to prepare the enzymatic assay system, we used 0.1 mg mL^−1^ of enzyme preparation, 4∙10^−4^ M NADH, 5.4∙10^−4^ M FMN, and 0.0025% tetradecanal solutions. The assay was performed in 0.05 M phosphate buffer, pH 6.8, at 25 °C.

The enzymatic assay system is based on the following coupled enzymatic reactions.

$$NADH+FMN\overset{NADH:FMN-oxidoreductase}{\to }FMN\cdot H^{-}+NAD^{+}$$

(reaction 1)

$$FMN\cdot H^{-}+RCHO+O_{2}\overset{luciferase}{\to }FMN+RCOO^{-}+H_{2}O+h\nu$$

(reaction 2)

The bioluminescence intensity was measured with biochemiluminometers TriStar LB 941 (Berthold technologies, Bad Wildbad, Germany) and Luminoskan Ascent (Thermo Electron Corporation, Solon, OH, USA).

Toxic effect of F10-12 on bioluminescence of enzymatic assay system was characterized by relative bioluminescence intensity, *I^rel^*:

*I^rel^* = *I_F_/I_contr_* (1)

where *I_contr_* and *I_F_* are maximal bioluminescence intensities in the absence and presence of F10-12, respectively.

**Figure 7:**
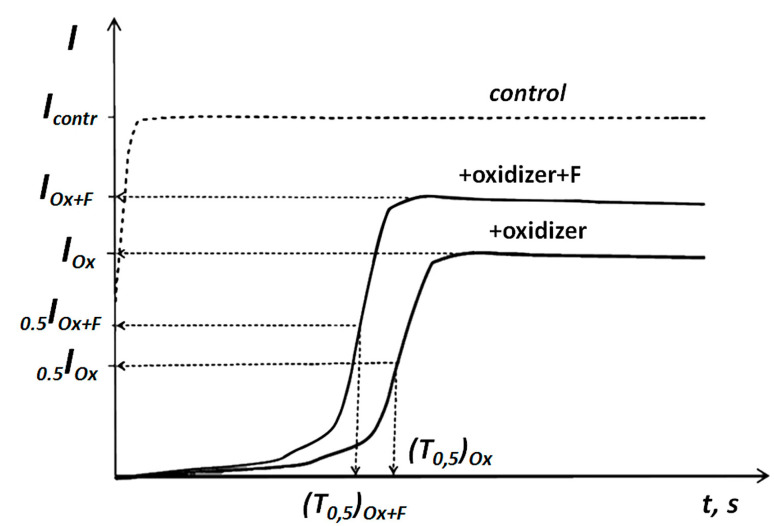
Bioluminescence kinetics of enzymatic assay in a solution of model oxidizers (*Ox*) and fullerene derivatives (*F*) [33].

In order to study antioxidant properties of F10-12, we exposed the bioluminescence assay system to model oxidizers (*Ox*)—potassium ferricyanide and 1,4-benzoquinone; *I_contr_* and *I_Ox_* measured bioluminescence intensity as shown in Figure 7. Effective concentrations of the model oxidizers inhibiting bioluminescence intensity by 50%, *EC_50_*, were determined, Figure 7. The *EC_50_* values were 10^−5^ M and 10^−4^ M in solutions of 1,4-benzoquinone and K_3_[Fe(CN)_6_], respectively. The values are close to those determined earlier [44,46].

Antioxidant activity of F10-12 was evaluated in the solutions of model oxidizers. The values of *EC_50_* of the oxidizers were used in these experiments. Concentration range of F10-12 that inhibited the bioluminescence intensity to less than 10% was preliminary determined and used in the experiments to exclude the peculiar toxic effects of F10-12.

In order to characterize changes of general toxicity in the oxidizer solutions under the addition of F10-12, the antioxidant coefficients *I^rel^_Ox_* were determined as follows:

*I^rel^_Ox_* = *I_Ox+F_/I_Ox_* (2)

where *I_Ox_*, *I_Ox+F_* are bioluminescence intensities in oxidizer solutions in the absence and presence of F10-12, respectively, Figure 7. Values of *I^rel^_Ox_* were determined at different concentrations of F10-12.

In order to characterize changes in oxidative toxicity in the oxidizer solutions under the fullerenol exposure, we used the antioxidant coefficients, *T^rel^_Ox_*:

*T^rel^_Ox_* = (*T_0.5_*)*_Ox_*/(*T_0.5_*)*_Ox+F_* (3)

where ${\left(T_{0.5}\right)}_{Ox}$ and ${\left(T_{0.5}\right)}_{Ox+F}$ are bioluminescence induction periods in the oxidizer solutions in the absence and presence of F10-12, respectively (Figure 7). The *T^rel^_Ox_* values were determined and plotted vs. F10-12 concentrations.

Values of *I^rel^_Ox_* > 1 or *T^rel^_Ox_* > 1 revealed a decrease in general or oxidative toxicities under the exposure to F10-12, i.e., antioxidant activity of F10-12 in solutions of oxidizers. Values of *I^rel^_Ox_* ≈1 or *T^rel^_Ox_* ≈1 revealed the absence of the fullerenol effects.

The SD-values for *I^rel^, I^rel^_Ox_* or *T^rel^_Ox_* did not exceed 0.15, 0.16 and 0.2, respectively (GraphPad Prism 8 (GraphPad Software, San Diego, CA, USA)). The data for the *I^rel^, I^rel^_Ox_,* or *T^rel^_Ox_* processing were obtained over two-three parallel experiments with five samplings from all solutions.

It should be noted that all experiments with “colored” solutions of fullerenol F10-12 and excluded the effect of “optic filter” [87] and this effect did not skew the results of the toxicological measurements. Optical density of solutions analyzed did not exceed 0.1 in the wavelength region of bioluminescence emission.

### 3.3. Luminol Chemiluminescence Assay

Luminol was obtained from Sigma-Aldrich (Burlington, MA, USA), 3% solution of H_2_O_2_ from Tula Pharmaceutical Factory (Tula, Russia), and potassium hydroxide from Khimreactiv (Nizhny Novgorod, Russia).

Stock luminol solution (10^−2^ M) was prepared as follows: luminol powder was dissolved in 5 mL in KOH (Khimreactiv, Nizhny Novgorod, Russia) and then 5 mL of distilled water was added. The 5.4·10^−5^ M alkaline luminol solution was applied to measure the chemiluminescence signal.

The chemiluminescence luminol reaction was initiated by 1.8·10^−4^ M K_3_[Fe(CN)_6_]; the maximum chemiluminescence intensity was determined. All chemiluminescence measurements were conducted in 10–15 replicates using the biochemiluminometer Luminoskan Ascent (Thermo Electron Corporation, Solon, OH, USA) with an injector system. SD values did not exceed 0.1, GraphPad Prism 8 (GraphPad Software, San Diego, CA, USA).

Initially, the dependence of chemiluminescence intensity on the concentration of H_2_O_2_ was determined; it was used as a calibration dependence in the following experiments to evaluate concentrations of peroxide compounds in the solutions of F10-12. Peroxides were considered as the constituents of the ROS group. 

The ROS content was studied in the bioluminescence assay system in the presence of F10-12 and/or 1,4-benzoquinone. Registration of chemiluminescence signal was provided after bioluminescence signal in the bioassay system.

All experiments with solutions of fullerenol F10-12 excluded the effect of “optic filter”, and this effect did not skew the results of ROS measurements. Optical density of solutions analyzed did not exceed 0.1 in the wavelength region of the chemilunescence light emittitance.

The ROS content was plotted vs. concentration of F10-12.

### 3.4. Statistical Processing

In order to reveal correlations between the bioluminescence signal and ROS concentrations, we analyzed a statistical dependence between the rankings of two variables [88]; correlation coefficients *r* were calculated.

Statistical processing of the results of chemiluminescence and bioluminescence assays was carried out; the *p* values were calculated with GraphPad Prism 8 (GraphPad Software, Inc., San Diego, CA, USA) using ANOVA. The *p* values were assessed by Kruskal–Wallis test of two independent sample distributions.

## 4. Conclusions

Our current paper studies the biological activity of a water-soluble fullerene derivative with a low number of oxygen groups (10–12) on the surface of its carbon cage. This compound was analyzed within a series of fullerenols with different numbers of oxygen groups. We compared toxicity and antioxidant activity of F10-12 to those of homologous fullerenols with higher number of oxygen groups. It should be noted that we aimed to elucidate the physicochemical processes underlying the toxic and antioxidant effects of F10-12 and chose the simplest bioassay based on a bioluminescent system of coupled enzyme reactions.

Low-concentration activation by F10-12 was not confirmed statistically in the bioassay system under standard experimental conditions; nevertheless, we successfully validated it under the conditions of an artificial oxidative load. Conditions of oxidative load denoted a presence of an oxidizer that suppresses bioluminescence intensity by 50%. The latter conditions were applied to study antioxidant activity of F10-12. Antioxidant coefficients *I^rel^_Ox_* and *T^rel^_Ox_* were determined in a wide low-concentration range of F10-12. These coefficients were calculated using such kinetic parameters such as bioluminescence intensity (*I*) and induction period (*T*), respectively, and were attributed to a change in general (polar/apolar + redox interactions) and oxidative (redox interactions only) toxicities in the oxidant solutions under the addition of fullerenol. Antioxidant coefficients *I^rel^_Ox_* and *T^rel^_Ox_* were higher in organic oxidizer solutions than compared to inorganic ones; this highlights the importance of hydrophobic interactions for redox transformations in the complex solutions under study.

The comparison of toxicity and antioxidant parameters of F10-12 to those of fullerenols F24-28 and F40-42 did not reveal a simple dependency on the number of oxygen groups: fullerenol F24-28 demonstrated lowest toxicity and highest antioxidant activity. It is likely that the higher efficiency of the fullerenol–solvent interactions of F24-28 and its related solubility in water [31] affects the properties of this fullerenol. This result contributes to the predictive criteria for selection of fullerene derivatives of optimal reactivity, which is highly important for biomedical applications.

In future studies, haemotoxicity and cytotoxicity tests [26,89,90,91,92,93] would assist in improving our understanding of the effects of fullerene oxygen derivatives on biological systems with ranging complexities.

Reactive oxygen species (ROS) were considered as active particles responsible for inhibiting (toxic) and activating (antioxidant) effects in the bioassay system. We found that both effects are concerned with a decrease in ROS content under the addition of the fullerenol: noticeable ROS decay results in toxic effect, while slight ROS decay in the solutions of the model oxidizer (1,4-benzoquinone) mitigates the toxic oxidizer’s effect revealing an antioxidant property of the fullerenol. Further theoretical simulations could likely explain the relations between the fullerene’s structure and its attendant redox activity.

We should emphasize that both the lack and an excess of ROS can produce an analogous deleterious effect. Hence, our results reveal a complexity of ROS effects in the enzymatic assay system.

## Figures and Tables

**Table 1:** Abbreviations for fullerenols C_60,70_O_y_(OH)_x_.

x + y	Abbreviation
10–12	F10-12
24–28	F24-28
40–42	F40-42

**Table 2:** Range of active concentrations and maximal values of *I^rel^_Ox_* for fullerenols in model oxidizer solutions of 1,4-benzoquinone and K_3_[Fe(CN)_6_].

Fullerenols	1,4-benzoquinone		K_3_[Fe(CN)_6_]	
	Active Concentrations, g L^−1^	Maximal Value of *I^rel^_Ox_*	Active Concentrations, g L^−1^	Maximal Value of *I^rel^_Ox_*
F10-12	10^–5^–4·10^−4^	1.38	-	1.0
F24-28 [33]	10^–18^–10^−10^	1.44	10^−18^–10^−4^	1.2
F40-42 [33]	10^−20^–10^−3^	1.44	-	1.0

**Table 3:** Range of active concentrations and maximal values of *T^rel^_Ox_* for fullerenols in model oxidizer solutions of 1,4-benzoquinone and K_3_[Fe(CN)_6_].

Fullerenols	1,4-benzoquinone		K_3_[Fe(CN)_6_]	
	Active Concentrations, g L^−1^	Maximal Value of *T^rel^_Ox_*	Active Concentrations, g L^−1^	Maximal Value of *T^rel^_Ox_*
F10-12	10^−13^–5·10^−5^	1.2	-	1.0
F24-28 [33]	10^−18^–10^−4^	1.9	10^−18^–10^−6^	1.3
F40-42 [33]	-	1.0	-	1.0

## Data Availability

Not applicable.

## Supplementary Materials

The following are available online at https://www.mdpi.com/article/10.3390/ijms22126382/s1.

## Abbreviations

EC_50_	effective concentration of oxidizers or fullerenols inhibited bioluminescence intensity by 50%
F10-12	C_60,70_O_x_(OH)_y_, where x + y = 10–12, y—even
F24-28	C_60,70_O_x_(OH)_y_, where x + y = 24-28
F40-42	C_60,70_O_x_(OH)_y_, where x + y = 40-42
FMN	flavinmononucleotide
NADH	nicotinamide adenine dinucleotide, disodium salt, reduced
ROS	reactive oxygen species
