# Supplementary figures and images for: Toxicity and Antioxidant Activity of Fullerenol C60,70 with Low Number of Oxygen Substituents
Source: Int J Mol Sci. 2021 Jun 15;22(12):6382. doi: 10.3390/ijms22126382 (PMC8232284; doi:10.3390/ijms22126382)

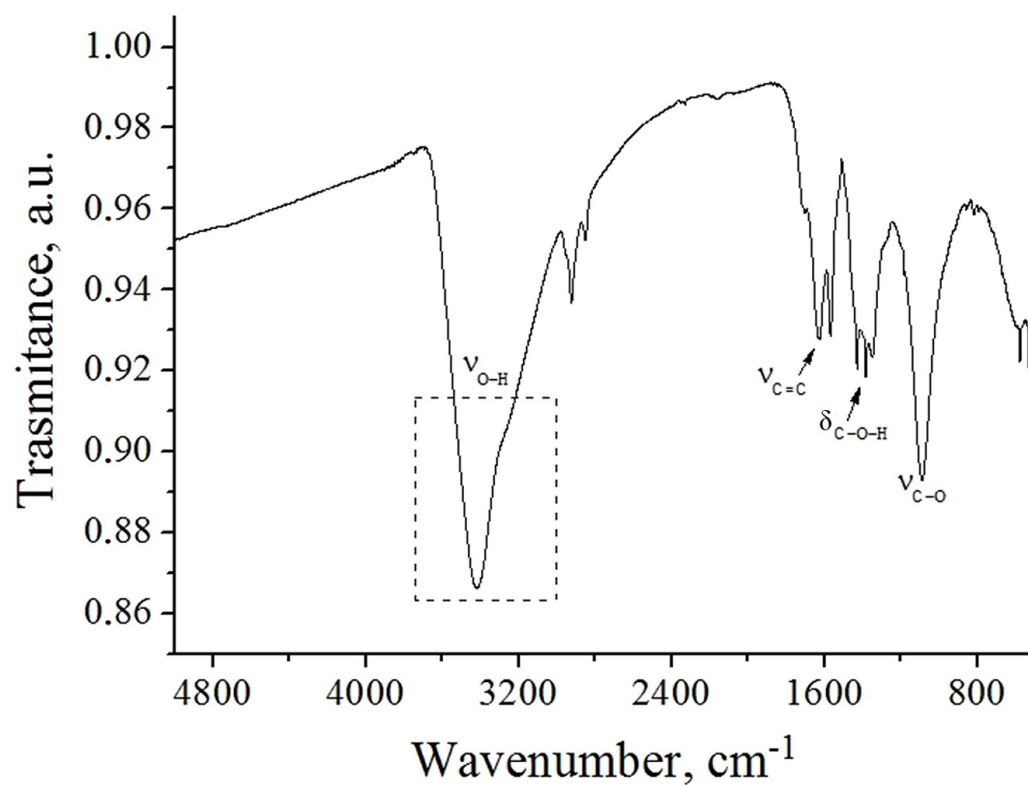

Figure S1: IR spectra of fullereneol F10-12.

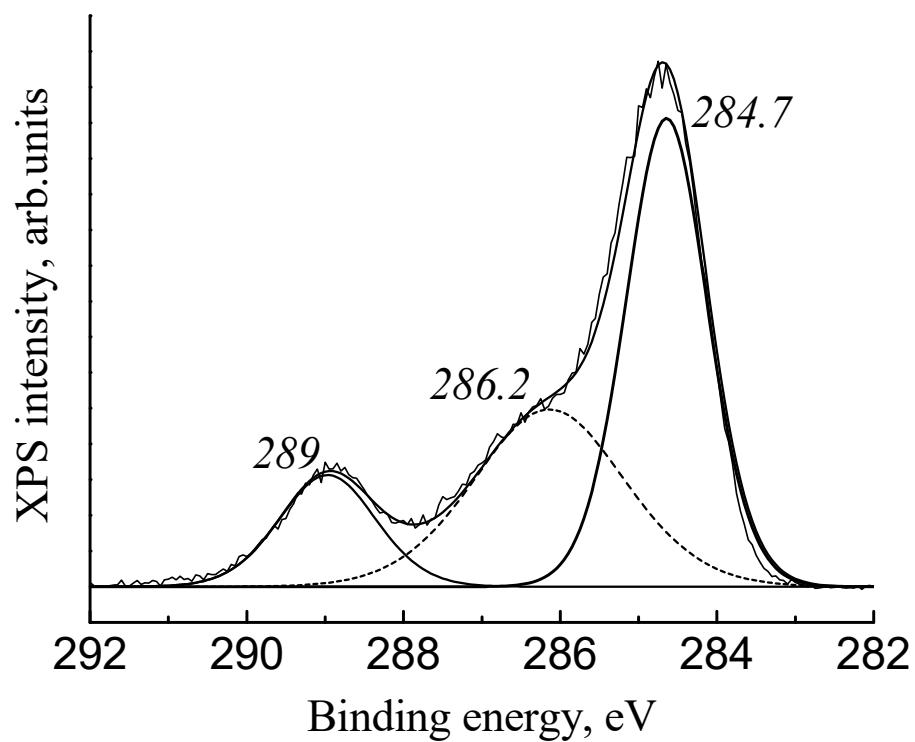

Figure S2: XPS of fullereneol F10-12 (C1s line).

Supplement: Supplementary file 1 [file ijms-22-06382-s001.zip › ijms-1242051-supplementary.pdf]
